# Isolation, Structure Elucidation, and Bioactivity Evaluation of Two Alkaloids From *Piper chaba* H. Stem: A Traditional Medicinal Spice and Its Chemico‐Pharmacological Aspects

**DOI:** 10.1002/fsn3.4585

**Published:** 2024-11-22

**Authors:** Shabiba Parvin Shandhi, Fahmida Tasnim Richi, Safaet Alam, Kutub Uddin Ahamed, Nazim Uddin Emon, Najneen Ahmed, Chuxiao Shao, Shuanghu Wang, Peiwu Geng, Abdullah Al Mamun

**Affiliations:** ^1^ Fiber and Polymer Research Division, BCSIR Dhaka Laboratories Bangladesh Council of Scientific and Industrial Research (BCSIR) Dhaka Bangladesh; ^2^ Department of Pharmaceutical Chemistry, Faculty of Pharmacy University of Dhaka Dhaka Bangladesh; ^3^ Chemical Research Division, BCSIR Dhaka Laboratories Bangladesh Council of Scientific and Industrial Research (BCSIR) Dhaka Bangladesh; ^4^ Pharmaceutical Sciences Research Division, BCSIR Dhaka Laboratories Bangladesh Council of Scientific and Industrial Research (BCSIR) Dhaka Bangladesh; ^5^ Department of Pharmacy, Faculty of Science and Engineering International Islamic University Chittagong Chittagong Bangladesh; ^6^ Department of Pharmacy East West University Dhaka Bangladesh; ^7^ Key Laboratory of Joint Diagnosis and Treatment of Chronic Liver Disease and Liver Cancer of Lishui, Central Laboratory of The Lishui Hospital of Wenzhou Medical University The First Affiliated Hospital of Lishui University, Lishui People's Hospital Lishui Zhejiang China

**Keywords:** antidiarrheal, antimicrobial, Chabamide, Chingchengenamide, Chuijhal, NMR, *Piper chaba*, spice, traditional medicine

## Abstract

Bangladesh is endowed with an abundance of excellent medicinal plant resources. A well‐known traditional medicinal plant *Piper chaba* H. from the Piperaceae family is rich in bioactive phytochemicals that have antidiarrheal, antimicrobial, analgesic, antioxidant, anticancer, and cytotoxic effects. This plant is locally known as “Chuijhal,” and the stem is used as spices. In the current research program, the stems of the *P. chaba* plant were selected and its chemical and biological investigations such as antidiarrheal, antimicrobial, and analgesic effects were performed. Moreover, docking models were accomplished by exploiting PyRx‐Virtual Screening software and implied that isolated compounds of *P. chaba* exert different pharmacological activity by inhibiting their targeted receptors. Phytochemical investigations revealed the isolation of Chingchengenamide A, a relatively rare alkaloid from the stems of *P. chaba*. Another alkaloid Chabamide I which is a piperine dimer was also isolated. Their structures were confirmed by comparing these compounds' spectral data (^1^H and ^13^C NMR) with their previously published spectral data. Antidiarrheal activity shows a percent reduction of diarrhea by 46.67% and 40%, respectively, for Chabamide I and Chingchengenamide A (at 20 mg/kg b.w.) compared with an 80% reduction by standard loperamide. Similarly, the percent reduction of writhing was 53.06% and 42.86%, respectively, for Chabamide I and Chingchengenamide A at similar doses compared with an 80% reduction by diclofenac sodium considered as standard. Both the alkaloids showed auspicious outcomes against test microorganisms during disk diffusion antimicrobial assay. Molecular docking and ADME/T analysis of the alkaloids also validate a potent pharmacological basis for the traditional utilization of *P. chaba* in treating diarrhea, pain, and microbial infection. These results emphasize the need to investigate *P. chaba* as a potential source of natural therapies for common health issues, laying the foundation for future research.

## Graphical abstract

The 1H‐NMR spectrum (400 MHz, CDCl3) of compound 1. The 13C NMR spectrum (100 MHz, in CDCl3) of compound 1. Structure of compound 1 as Chabamide I. Purity assessment of compound 1 in TLC plate.

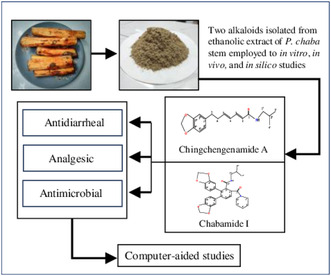

## 1 Introduction

Herbal medicines have been vital to the advancement of human civilization (Hosseinzadeh et al. 2015). A wide range of plants with therapeutic qualities are referred to as medicinal plants. Compounds from these plants are abundant and can be utilized to create new drugs (Rasool Hassan 2012). Due to a lack of access to modern healthcare services, and the effectiveness of traditional medicines, herbal medicines offer developing nations a cheap alternative (Mesa et al. 2021; Sultana et al. 2022). For 80% of people globally, traditional medicine is their major source of health care, according to the World Health Organization (WHO) (Atanasov et al. 2021; Balandrin, Kinghorn, and Farnsworth 1993; Riaz et al. 2012).

In traditional medicine and folk medicine, secondary plant metabolites (such as alkaloids, phenolics, and carotenoids) play a significant role in the treatment of various illnesses (Bachhar et al. 2024a; Bachhar et al. 2024b). Several plant‐derived secondary metabolites, the human body is affected by these substances pharmacologically such as cytotoxicity, hepatoprotection, antioxidants, and antidepressants, have been shown to benefit the liver (Jiko et al. 2024). This is because of their exceptional biological activity, which includes tannins, phenolic compounds, alkaloids, cyclitol (Al‐Suod et al. 2019), and flavonoids (Duraipandiyan, Ayyanar, and Ignacimuthu 2006). Because of this, it's becoming more and more popular to extract, separate, and purify secondary metabolites from plants. A variety of separation techniques are used in this process (Al‐Suod et al. 2019).

Diarrhea, marked by heightened liquid excretion and abdominal distress, presents a significant health burden (Tadesse et al. 2014). Especially prevalent in impoverished regions, it stands as a primary contributor to malnutrition and mortality (Zhao et al. 2018), accounting for over 4,800,000 child fatalities annually (Agbor et al. 2014). Despite the availability of basic remedies such as oral saline and antibiotics, diarrhea persists as a formidable health challenge. Plant extracts have drawn interest because of their potential to reduce the symptoms of diarrhea by promoting the absorption of water, reducing the loss of electrolytes, and modifying the movement of food through the gastrointestinal tract (Agbor, Léopold, and Jeanne 2004; Shifah et al. 2020).

A wide range of detrimental consequences, including destruction, deterioration, and various exudative reactions, is caused by inflammation, which is characterized by a multitude of complex pathways and heterogeneous mediators (Medzhitov 2008). Pain and inflammation are treated by using analgesic pharmaceuticals, which include opioids like morphine and fentanyl, nonsteroidal anti‐inflammatory drugs (NSAIDs), and newer forms of treatment such as gabapentin, carbamazepine, and ketamine. Moreover, glucocorticoids elicit a complex reaction through receptor binding, which induces elevated levels of anti‐allergic proteins (e.g., IL‐1 antagonist) and simultaneous downregulation of activated transcription factors (e.g., NF‐κB) (Barnes 1998). Through their pharmacological action, NSAIDs obstruct the activity of cyclooxygenase enzymes (COX‐1 and COX‐2), which are essential for the manufacture of many inflammatory mediators. Despite the commercial availability of an assortment of NSAIDs, their protracted usage is associated with substantive side effects, notably gastrointestinal ulceration, liver toxicity, and renal impairment (Sostres et al. 2010). Consequently, there exists a discerning interest in exploring novel phytochemical compounds as potential alternatives, as espoused by Liu (2007).

Infectious diseases represent a distinct class of clinical maladies, accounting for a staggering 25% of hospital admissions and 20% of annual mortality rates (Thabit, Crandon, and Nicolau 2015). Interestingly, bioactive phytoconstituents have shown encouraging therapeutic potential in the fight against these illnesses (Rios and Recio 2005; Barnes and Heinrich 2004). Even with the abundance of commercially accessible antibacterial medications, new therapeutic strategies are still needed because of the growing threat of resistance patterns and the resulting increase in mortality (Roberts et al. 2010). Furthermore, the mounting healthcare expenditures and mortality rates underscore the imperative for the discovery of novel antimicrobial agents endowed with diminished side effects, aimed at mitigating the burden of mortality (Roberts et al. 2009). With a variety of antibacterial properties, plants and their products can be considered a superior substitute. Various phytochemicals, such as neophytadiene, phytol, stigmasterol, squalene, are proven antimicrobial agents present in plant extract (Bachhar et al. 2024a; Bachhar et al. 2024b).

The Piperaceae family is well known for its medicinal importance. Numerous diseases, including fever, headache, diarrhea, joint pain, boils, scabies, and digestive issues, are treated with the consumption of piper species (Tsai et al. 2005; Chakraborty and Shah 2011; Sharkar et al. 2013; Umoh et al. 2013; Aziz, Hama, and Alam 2015). Additionally, they work well in the management of breathing conditions (Mohamad et al. 2011). The species have hepatoprotective and gastrointestinal properties (Kumar et al. 2010). Additionally, extracts from Piper species have been shown to exhibit antioxidant and anti‐inflammatory attributes (Vaghasiya, Nair, and Chanda 2007; Sarkar et al. 2008; Sarkar et al. 2013). This species exhibits antidiabetic, antihypertensive, immunoprotective, neuroprotective, and anticarcinogenic effects (Shandhi 2024).

**FIGURE 1:**
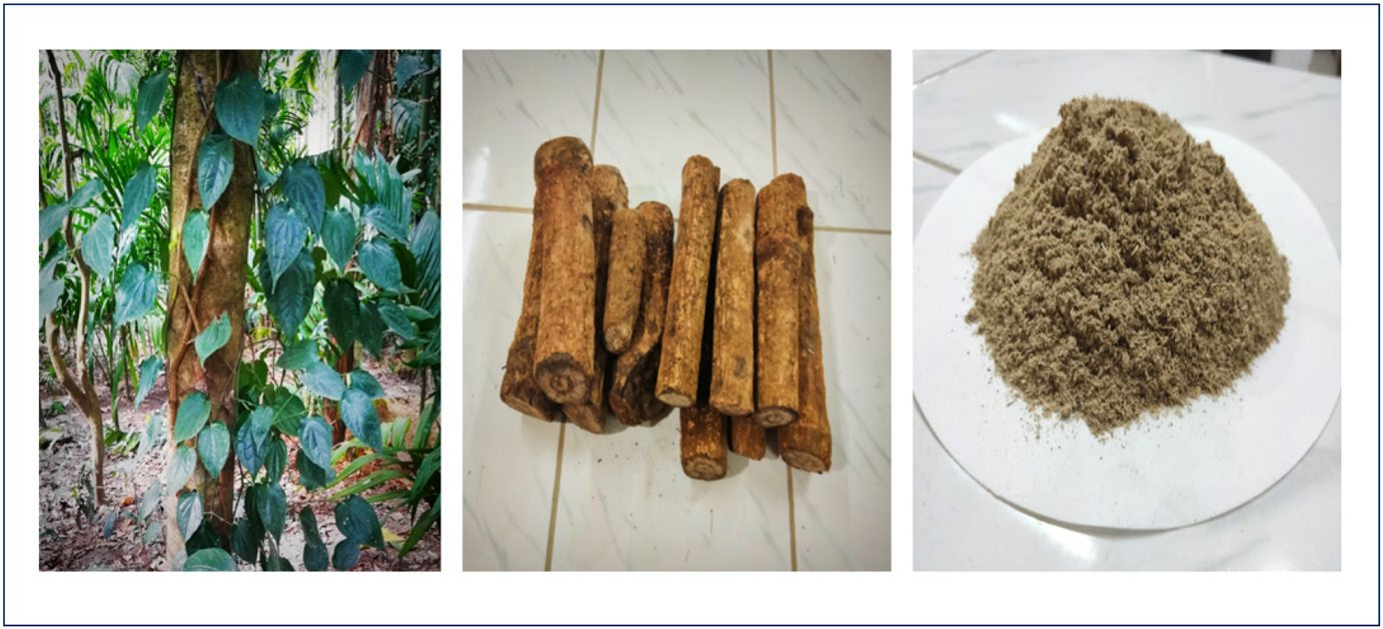
*P. chaba* H. (Chuijhal) plant, stems, and powdered stem.

A member of the Piperaceae family, the flowering vine *Piper chaba (P. chaba)* (Figure 1) is reported to be grown in Indonesia, Singapore, Sri Lanka, Bangladesh, and some parts of India, including Tripura, Kerala, and West Bengal (Joshi et al. 2024; Haque et al. 2018). The medicinal plant is known as Chuijhal in Bangladesh, Tripura (India), and West Bengal (India) (Islam et al. 2020). Though the roots have a stronger scent than the stems, they are nonetheless costlier than the stems in Bangladesh. The leaves, stems, and seeds of medicinal piper species are widely grown for their strong scent and acridity taste, which makes them valuable spices.

In Bangladesh, Chuijhal is a special kind of traditional medicinal plant. Other than this, *P. chaba* is utilized in many foods and for other purposes and does have nutritional and antioxidant qualities. Additionally, *P. chaba* is said to be edible and nontoxic (Prajapati and Kumar 2023). The antioxidant activity of its leaf extract is prominent and important fatty acids have been found in its leaf extract (Shandhi 2024). Specialized carbamide piperine dimer and alkaloids with pharmacological and antibacterial qualities are found in the stems of *P. chaba* (Rukachaisirikul et al. 2002; Alam et al. 2024). Many studies demonstrate the numerous powerful pharmacological characteristics of piperine, including its antipyretic (Sabina et al. 2013), anticancer (Manayi et al. 2018), antitumor, anti‐analgesic (Dhargawe et al. 2021), antidiarrheal (Alam et al. 2020), anticoagulant (Zayed et al. 2020), antimicrobial (Hikal 2018), and antioxidant (Mittal and Gupta 2000). The anti‐inflammatory properties of piperine are demonstrated (Mujumdar et al. 1990). The activation of the TRPV1 receptor causes a burning sensation (Dong et al. 2019; Correa et al. 2010; Chen et al. 2013). Chemicals extracted from *P. chaba* H.'s fruits and roots (Morikawa et al. 2004) have pharmacological activity against various illnesses. In rats with liver damage generated by lipopolysaccharide and D‐galactosamine (D‐GalN), an aqueous acetone extract of *P. chaba* fruits exhibits hepatoprotective properties (Matsuda et al. 2008; Matsuda et al. 2009; Morikawa 2010).

The present investigation emphasizes the pharmacological analysis, separation of the bioactive substances from *P. chaba* H. stems, and structural clarification using NMR spectroscopy. Furthermore, the analgesic, antimicrobial, and antidiarrheal qualities of isolated phytochemicals were investigated. Additionally, the activities of the phytochemicals were predicted and supported through the application of molecular docking. Understanding the mechanism behind the potential pharmacological action in the drug design paradigm is a crucial step. We have structurally exposed the active phytoconstituents and diverse pharmacological potential including antidiarrheal, analgesic, and antimicrobial activity. In this study, we utilized advanced computer‐aided drug design techniques to establish the exact antidiarrheal, analgesic, and antimicrobial targets that these phytochemicals bind to exert pharmacological action. The fact that *P. chaba* is a safe food and spice means that it will have a significant impact on people's health concerns. Synthetic drugs have several adverse effects, so research on medicinal plants will help to discover safe drugs for mankind. That's why this area is given a big interest.

## 2 Materials and Methods

### 2.1 Plant Source

Forty stems of *P. chaba* (Locally known as Chuijhal) ranging in age from 4 to 5 years (mean age 4.5 years) and weighing between 200 g and 300 g (mean weight 250 g) were collected from Borobari, Kurigram, the northern part of Bangladesh at 25.8133° N, 89.6483° E coordinate in September 2023. A taxonomist named Sajib Rudra from the Botany Department, University of Chittagong, Bangladesh, determined the plant's taxonomy. Next, the plant (accession no.: CTGUH SR‐7928) was placed in storage for more examination.

### 2.2 Chemicals and Instruments

All solvents and reagents used during the investigation were procured by Merck (Germany) and BDH (England). The solvents, which were commercial‐grade, were distilled before use. Köfler type melting point apparatus was used for melting point measurement. Shimadzu UV‐1601, a UV spectrophotometer, and a Shimadzu IR prestige‐21(FT‐IR) spectrometer were used for UV and IR. NMR was taken in CDCl_3_ and CD_3_OD on a BRUKER NMR DPX‐400 MHz instrument (BCSIR, Dhaka, Bangladesh) at 400 MHz for protons and 100 MHz for carbons using TMS as the internal standard. All NMR spectra were obtained using the standard Bruker software. The UV lamp Mineralight device, multiband UV‐254/366 nm obtained from UVP Inc., USA, was used for TLC plate visualization. Column chromatography (CC) was carried out over silica gel (230–400 mesh; ASTM, Merck), and TLC was carried out with silica gel 60 pre‐coated plates F‐254 (Merck).

### 2.3 Test Microorganisms

To conduct the antimicrobial assay, a selection of five gram‐positive bacteria ( *Bacillus megaterium* [ATCC 25918], *Staphylococcus aureus* [ATCC25923], *Bacillus cereus* [clinical isolates], *Bacillus subtilis* [ATCC6633], and *Sarcina lutea* [clinical isolates]) and seven gram‐negative bacteria ( *Salmonella typhi* [clinical isolates], *Escherichia coli* [ATCC25922], *Salmonella paratyphi* [clinical isolates], *Pseudomonas aeruginosa* [ATCC27833], *Vibrio mimicus* [clinical isolates], *Shigella dysenteriae* [clinical isolates], and *Vibrio parahemolyticus* [clinical isolates]) were employed.

### 2.4 Test Animal Models

To carry out the in vivo experiment, Swiss albino mice aged between 4 and 5 weeks, of both genders, were obtained from the Animal Resource Branch of the International Centre for Diarrheal Diseases and Research, Bangladesh (ICDDR, B) (the size of the animal population, *n* = 5). The mice were housed in standard polypropylene cages with a 12‐h light–dark cycle. Additionally, optimal conditions such as a controlled room temperature of 24°C ± 2°C and a relative humidity of 60%–70% were upheld. They were provided with ICDDR, B‐formulated rodent food, and water ad libitum. The research protocol was approved by the Animal Ethics Committee of the State University of Bangladesh, Dhaka (2023‐01‐04/SUB/A‐ ERC/003) before initiating the study. Throughout the experiments, strict adherence to guidelines for the use and care of laboratory animals was ensured. Recognizing the sensitivity of mice to environmental variations, they were acclimatized to the experimental conditions for a minimum of 3–4 days before the commencement of the experiment. Furthermore, all ethical protocols and regulations were strictly adhered to during the design and execution of the research experiments. At the end of the experiment, an anesthesia overdose (Ketamine HCl [100 mg/kg] and Xylazine [7.5 mg/kg]) through the intraperitoneal route was given to the mice models followed by euthanasia, following the previously delineated protocols established by Zimmermann (1983) and Davis (2001) (Alam et al. 2021; Davis 2001; Zimmermann 1983). All tests were carried out in abiding with the guidelines for the care and use of laboratory animals, as approved by the institutional ethical committee.

### 2.5 Extraction and Isolation

Plant samples were collected, cleaned, and dried for several days before being processed into a coarse powder (Figure 1). Following that, the 750 g of powdered stem samples was soaked in ethanol for 10–15 days at room temperature while being periodically shaken. The extracts of the stem samples were filtered by cotton and filter papers. The excess solvent was then removed using a rotary evaporator. After the solvent evaporation, a solid residue (15 g) was recovered and refrigerated for later use.

The extract was then soaked in water and fractionated using several solvents. *n*‐hexane (HEX), dichloromethane (DCM), and ethyl acetate (EAC) with increasing polarity, solvent–solvent partitioning was carried out by Kupchan's method, which was updated by VanWagenen et al. (1993). The fractionates were then evaporated to dryness (Rolta et al. 2022; Rolta et al. 2020). The amount of different fractionates was *n*‐hexane (3.5 g), DCM (6.0 g), ethyl acetate (2.44 g), and aqueous (2.84 g).

**FIGURE 2:**
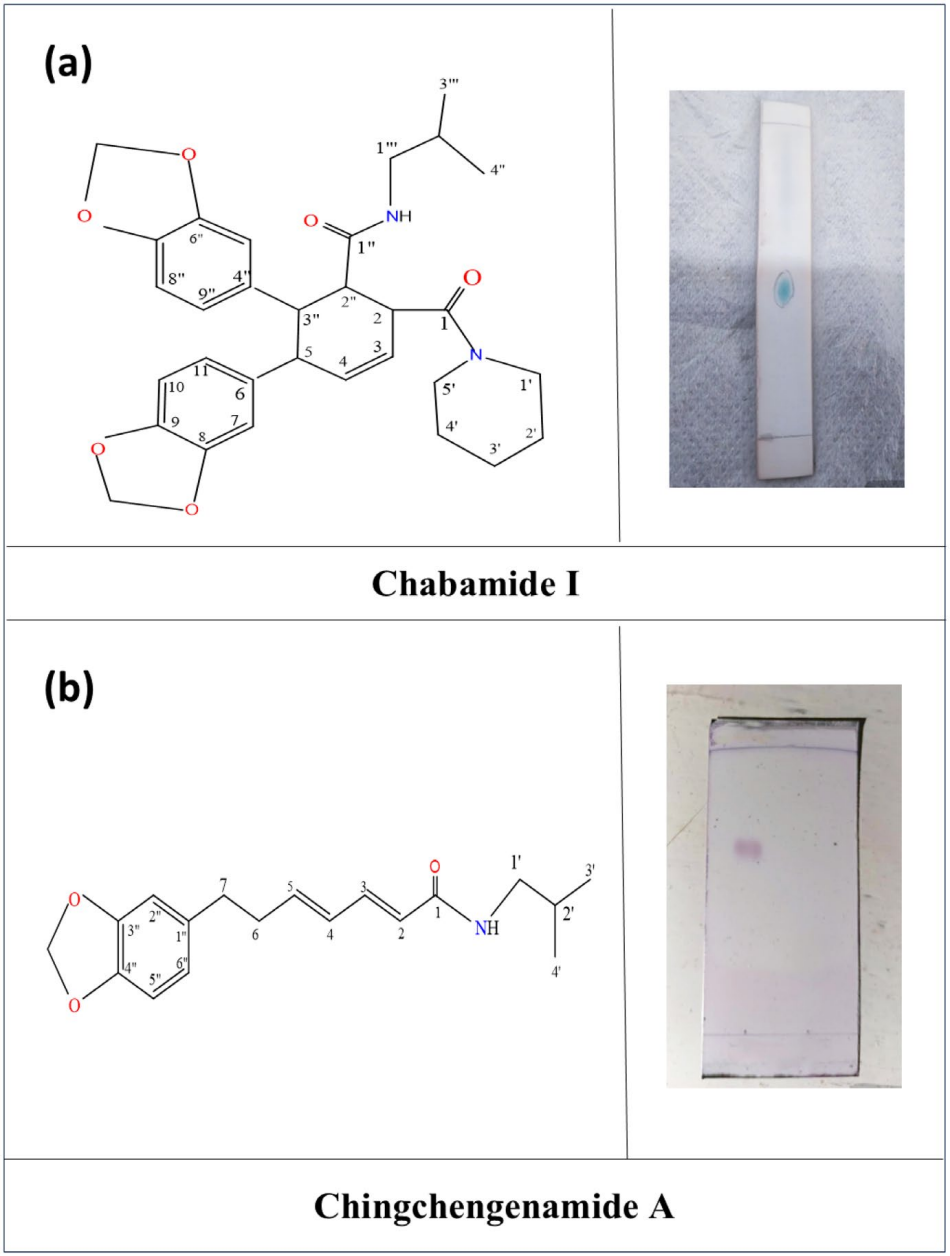
(a) Structure of the compound 1 as Chabamide I along with purity assessment of Chabamide I in TLC plate; (b) Structure of the compound 2 as Chingchengenamide A along with purity assessment of Chingchengenamide A in TLC plate.

The DCM extract was taken to TLC analysis to see the presence of phytochemicals in the extract. Then, the DCM (~ 6.0 g) part was subjected to CC and eluted with nonpolar to polar solvents. The fractions were collected and then repeated the process to collect different fractions. The fractions were subjected to preparative TLC to purify the compounds and characterized by the NMR spectroscopy method. Two compounds were isolated purely. Compound 1 (6 mg) was found in a white crystalline solid from 10% n‐hexane in DCM, and compound 2 (~8 mg) was found in a powdered solid from 5% EAC in DCM. The purity of compounds was detected by confirming a single spot in the TLC plate (Figure 2).

### 2.6 In Vivo Acute Oral Toxicity

Under standard laboratory circumstances, the oral toxicity test was done with the fixed‐dose method according to the “Organization for Environmental Control Development” standards (OECD: standards 420) (Van den Heuvel 1984; Rudra et al. 2020), and following the mice's oral administration of large doses (2000 mg/kg), some parameters were monitored over the next 72 h. Therefore, following oral administration of the ethanol‐soluble compounds, extract of *P. chaba*, no mortality, no behavioral alteration (sedation, excitability), and no allergic reaction were found. For the antidiarrheal activity investigation, dosages of 200 and 400 (mg/kg, b.w.; p.o.) have been chosen in consideration of the safe dosage adjustment concerning oral acute toxicity.

### 2.7 Antimicrobial Assay

#### 2.7.1 Disk Diffusion Test

To assess the antimicrobial efficacy of the compounds Chabamide I and Chingchengenamide A isolated from *P. chaba*, we employed the disk diffusion method by Huys, Huys, and D'haene, K. and Swings, J. (2002) with little modification. For the susceptibility assay, we selected three commonly utilized antibiotic agents available in the market: azithromycin, amoxicillin, and ciprofloxacin. The nutrient‐agar medium dishes were pre‐inoculated with the following sample disks, test bacterial strains, standard antibiotic disks, and control disks were delicately placed onto the agar surface. For a whole day, the plates were incubated at 37°C. Although the standards were employed at 30 μg/disk, isolated phytochemicals were measured at 100 μg/disk. Following incubation, the diameters of the clear zones surrounding each disk were meticulously measured to assess the antimicrobial activity.

### 2.8 Antidiarrheal Assay

#### 2.8.1 Castor Oil‐Induced Diarrhea Test

The antidiarrheal potential of the compounds Chabamide I and Chingchengenamide A isolated from *P. chaba* was scrutinized employing the castor oil‐induced method, as delineated by Shoba and Thomas (2001) with slight adjustments. In this investigation, mice were judiciously randomized into four cohorts, each consisting of six subjects. Before experimentation, the mice underwent an overnight fasting period with unrestricted access to water. Subsequently, 0.5 mL of castor oil was applied to induce diarrhea, with only those exhibiting symptomatic manifestations selected for further analysis.

Group I, designated as the control, received vehicle solutions comprising distilled water with 1% Tween‐80. Group II, the positive control or standard drug cohort, was subjected to loperamide, a recognized antimotility drug, at a dose of 5 mg/kg body weight. Groups III and IV, representing the test cohorts, received isolated phytochemicals individually at oral dosages of 10 and 20 mg/kg body weight, respectively. An additional 0.5 mL of castor oil was applied to all subjects 1 h after the initial application of test samples. Subsequently, individual mice were placed in enclosures with floors lined with transparent paper.

Throughout the observational timeframe, diverse parameters were meticulously monitored, encompassing the initiation of diarrhea, quantification of wet stools in terms of number and weight, and the cumulative number and weight of fecal output. The observation persisted until 4 h before the subsequent application of castor oil, with the calculated mean values being designated as the definitive results.

### 2.9 Analgesic Assay

#### 2.9.1 Acetic Acid‐Induced Writhing Test

The analgesic efficacy of the isolated compounds, Chabamide I and Chingchengenamide A, was assessed utilizing the acetic acid‐induced writhing test, following the methodology outlined by Ahmad et al. (2010). Groups I and II were administered tween‐80 (10 mL/kg; b.w, p.o) and acetylsalicylic acid (10 mg/kg; b.w, i.p.), respectively, serving as positive and negative controls. Conversely, Groups III and IV received individual alkaloids at doses of 10 and 20 mg/kg; b.w, p.o., respectively. Subsequently, an intraperitoneal injection of acetic acid solution (0.6% v/v) was administered. The frequency of writhing responses was meticulously recorded at 5‐min intervals post‐acetic acid injection and monitored over 25 min.

#### 2.9.2 Statistical Analysis

The statistical interpretation was presented in the format of mean ± standard error of the mean (SEM). The acquired values underwent a comparative analysis with those of the control group, and statistical significance was discerned (***p < 0.001, **p < 0.01, and *p < 0.05). This analysis was followed by a one‐way analysis of variance (ANOVA) accompanied by Dunnett's test for further validation. All statistical computations were meticulously executed utilizing GraphPad Prism Version 5.2 (San Diego, CA).

### 2.10 In Silico Studies

#### 2.10.1 Optimization of Geometry

In developing new drugs, using computer tools to forecast the characteristics of novel substances is becoming increasingly common. Biological features, including geometric patterns, thermodynamics, molecular orbital (MO), electrostatic potential (ESP), and spectral analysis, are easily predicted without costly experiments (Uzzaman et al. 2021). The online PubChem server (https://pubchem.ncbi.nlm.nih.gov/) is used to compile the original chemical composition of Chabamide I and Chingchengenamide A. The conformer with the highest stability, and the lowest energy was identified using the anticipated AMBER potential value obtained using the Gabedit software (Allouche 2011). The Gaussian 09 W software was utilized for all quantum chemistry computations, with the CAM‐B3LYP 6311G basis set of density functional theory (Lapointe and Weaver 2007). If the terms potentiality (μ), hardness (η), softness (S), and HUMO‐LUMO energy gap (ΔE) are mentioned, the following equations (Afrin et al. 2023) were used to determine them:

$$\mathrm{Gap}\;\left(\mathrm{\Delta E}\right)=\left[\text{εLUMO}–\text{εHOMO}\right]$$

$$\mu =\frac{\left[\text{εLUMO}+\text{εHOMO}\right]}{2}$$

$$\eta =\frac{\left[\text{εLUMO}–\text{εHOMO}\right]}{2}$$

$$S=\frac{1}{2\eta }$$

#### 2.10.2 Preparing Proteins, Docking Molecules, and Computing Interactions

The RCBS Protein Data Bank in PDB format is the source of the three‐dimensional crystal structures for prostaglandin‐2 (PDB ID: 6COX) (Kurumbail et al. 1996), aspirin‐acetylated human cyclooxygenase‐2 (PDB ID: 5F19) (Lucido et al. 2016), delta‐opioid receptor (PDB ID: 4RWD) (Fenalti et al. 2015), kappa opioid receptor (PDB ID: 6VI4) (Che et al. 2020), beta‐ketoacyl‐acyl carrier protein synthase III (PDB ID: 1HNJ) (Qiu et al. 2001), and antibacterial protein (PDB ID: 1AJ6) (Holdgate et al. 1997). The Discovery Studio Visualizer 2020 program eliminated all water molecules, unexpected chains, heteroatoms, undesirable chains, and co‐crystallized ligands. To eliminate any unwanted interactions between the medication and protein, the protein chain was energy reduced using the conjugate gradient technique using the Swiss‐PdbViewer (Version 4.1.0) program (Ciucx and Peitsrh Urctrophuresis 1997). PyRx Autodock vina has been utilized to assess the relationship between the isolated compounds of *P. chaba* and proteins. The grid box was generated in the box center with an active site. Finally, the Discovery Studio Visualizer 2020 was used to compute the nonbonded interaction and analyze the docking data. (Prajapati et al. 2022).

### 2.11 ADMET Prediction

When evaluating the safety and efficacy of a drug candidate as a therapeutic agent, attributes about absorption, distribution, excretion, metabolism, and toxicity (ADMET) are crucial. Because of this, medicinal chemistry and computational chemistry have emerged as the most effective methods for predicting ADMET features in recent decades. Two isolated and identified compounds of *P. chaba* are used for ADMET characteristics prediction using the ADMET SAR (http://lmmd.ecust.edu.cn/admetsar1) website.

## 3 Results

### 3.1 Isolated Phytochemicals

Compound 1 was a white crystalline solid (~6 mg); R_f_ = 0.65 (90% DCM: 10% HEX); ^1^H and ^13^C NMR(TMS) data were found similar to the previously published data in Table 1.

**TABLE 1:** ^13^C and ^1^H NMR spectral data of compounds 1 and 2 (100, 400 MHz, DMSO‐d_6_, and TMS).

Position no.	Compound 1		Compound 2	
	δ_c_	δ_H_ (*J* in Hz)	δ_c_	δ_H_ (*J* in Hz)
1	162.77	—	165.68	—
2	55.80	Overlapping (1H)	121.19	5.94 (1H, d)
3	122.29	4.14 (1H, dt)	141.02	7.19 (1H, dd)
4	130.44	6.05 (1H, ddd)	129.48	6.14 (1H, m)
5	55.93	Overlapping (1H)	142.63	6.14 (1H, m)
6	131.38	—	35	2.42 (2H, q)
7	111.69	6.28 (1H, br,s)	35	2.64 (2H, t)
8	147.92	—	—	—
9	149.10	—	—	—
10	107.95	6.56 (1H, d)	—	—
11	120.65	Overlapping (1H)	—	—
1′	46.93	3.85 (2H, m)	45.85	3.57 (2H, t)
2′	28.95	1.32–1.79 (2H, m)	29.71	1.4–1.8 (1H, m)
3′	28.65	Overlapping (2H)	24.65	0.92 (3H)
4′	28.73	Overlapping (2H)	24.65	0.92 (3H)
5′	41.25	3.85 (2H, m)	—	—
6′	—	—	—	—
1^″^	178.53	—	135.17	—
2^″^	38.3	3.15 (1H, t)	108.84	6.64 (1H, br,s)
3^″^	34.79	2.94 (1H, dd)	147.59	—
4^″^	54.68	—	145.74	—
5^″^	109.49	6.24 (1H, br,s)	108.17	6.72 (1H, d)
6^″^	146.49	—	119.03	6.60 (1H, d)
7^″^	143.15	—	—	—
8^″^	105.40	6.46 (1H, d)	—	—
9^″^	120.21	6.07 (1H, br,s)	—	—
1′′′	46.48	2.57 (2H, m)	—	—
2′′′	29.34	Overlapping (1H)	—	—
3′′′	20.13	0.92 (3H, d)	—	—
4′′′	19.89	0.92 (3H, d)	—	—
Methylene di‐oxy	100.92 101.06	5.93 (2H, d) 5.82 (2H, d)	100.8	5.90 (2H, s)

The ^1^H‐NMR spectrum (400 MHz, CDCl_3_) of compound 1 showed peaks at δ 4.14 (dt), 6.05 (ddd), 6.28 (br, s), 6.56 (br, s), 3.85 (m), 1.32–1.79 (m), 5.93 (d), 5.82 (d), 3.15 (t), 2.94 (dd), 6.24 (br, s), 6.46 (d), 6.07 (br, s), 2.57 (m), and 0.92 (d) ppm (Figure S1). The ^13^C‐NMR spectrum (100 MHz, CDCl_3_) of compound 1 showed main chemical shift at 162.77, 55.80, 122.29, 130.44, 55.93, 131.38, 111.69, 147.92, 149.10, 107.95, 120.65, 46.93, 28.95, 28.65, 28.73, 178.53, 100.92, 101.06, 41.25, 38.30, 34.79, 54.68, 109.49, 146.49, 143.15, 105.40, 120.21, 46.48, 29.34, 20.13, and 19.89 ppm (Figure S2).

Compound 2 was powdered solid (~8 mg); R_f_ = 0.36 (95% DCM: 5% EAC); ^1^H and ^13^C NMR (TMS) data were found similar to the previously published data in Table 1.

The ^1^H‐NMR spectrum (400 MHz, CDCl_3_) of compound 2 showed peaks at 5.94 (d), 7.19 (dd), 6.14 (m), 2.42 (q), 2.64 (t), 6.64 (br, s), 6.72 (d), 6.60 (d), 5.90 (s), 3.57 (t), 1.4–1.8 (m), and 0.92 ppm (Figure S3). The ^13^C‐NMR spectrum (100 MHz, CDCl_3_) of compound 2 showed main chemical shift at 165.88, 121.19, 141.02, 129.48, 142.63, 35, 135.17, 108.84, 147.59, 145.74, 108.17, 119.03, 100.8, 45.85, 29.71, and 24.65 ppm (Figure S4).

Compound 1 exhibited benzene ring proton signals at 6.28 (br,s), 6.56 (d), 6.24 (br,s), 6.46(d), and 6.07 (br,s) ppm in its ^1^H NMR spectrum (400 MHz, in CDCl_3_). At 6.56 ppm, the signals from the proton at location 10 and position 11 overlapped and the isopropyl methyl [(CH_3_)_2_CH‐] signals at δ 0.92 (6H, d) ppm. The signals at 1.32–1.79 ppm multiplets for protons of methylene (‐CH_2_‐) and the signal of the proton of C‐2′′′ position overlapped at 1.32–1.79 (m) ppm. Multiplets were obtained at 2.57 ppm from another methylene proton of the C‐1′′′ position. The two methylene di‐oxy protons produced doublet at 5.93 and 5.82 ppm.

The multiplets at 3.85 ppm were produced by the four protons attached to methylene(‐CH_2_‐) carbon (attached to nitrogen) at positions 1^⸍^ and 5^⸍^. The methine (‐CH‐) protons at positions 3 and 4 showed signals at 4.14 (dt) and 6.05 (ddd) ppm, which were downfield due to double bond carbon (deshielded), whereas the signals for 3° carbon protons of positions 2^″^ and 3^″^ were at 3.15 (t) and 2.94 (dd)ppm, which were upfield due to single bond carbon (shielded). At 3.85 ppm, the signal of two protons from 3° carbons 2 and 5 overlapped as multiplets. We observed a quadrupole widening for nitrogen atoms at 5.5 ppm, and the Lessaigne test also confirmed the presence of nitrogen.

The presence of two aromatic rings in compound 1 was explained by its ^13^C NMR spectrum (100 MHz, in CDCl_3_). In two benzene rings, there were six quaternary carbon atoms in total. Of these, four were oxygenated and produced signals farther downfield than the other carbon atoms in the rings. The signals for the quaternary carbons at positions 8, 9, 6^″^, and 7^″^ were found to be 147.92, 149.10, 146.49, and 143.15 ppm, respectively. The signals for the remaining two quaternary carbons were found to be 131.38 and 54.68 ppm. Methine (‐CH‐) carbons made up six more carbons of the two benzene rings; signals were seen at 111.69, 107.95, 123.7, 109.49, 105.40, and 120.21 ppm. Signals at 100.92 and 101.06 ppm for two benzene rings were detected that have two methylene di‐oxy (‐OCH_2_O‐) groups linked to them. Additionally, there were two indications for carbonyl groups at 162.77 and 178.53 ppm. Methylene (‐CH_2_‐) carbons attached to the nitrogen displayed signals at 46.48, 46.93, and 41.25 ppm. Signals were detected at 28.95, 28.65, and 28.73 ppm by three additional methylene carbons with the nitrogen ring.

Two ‐CH_3_ carbon signals at 20.13 and 19.89 ppm and one ‐CH‐ carbon signal at 29.34 ppm indicated the presence of an isopropyl [(CH_3_)_2_CH‐] group in compound 1. The last six carbon atoms were arranged in a ring with a double bond; two methine (‐CH‐) carbons in this ring were in the double bond contact and produced signals at 122.29 and 130.44 ppm. At 55.80, 55.93, 38.30, and 34.79 ppm, the signals for the remaining four tertiary carbons were detected, which were upfield than protons in methine (‐CH‐).

From the physical characteristics and spectral analysis (^1^H‐NMR and ^13^C‐NMR) data of compound 1, and comparing the previously published ^1^H‐NMR and ^13^C‐NMR spectral data of Chabamide I, the structure of compound 1 was established as Chabamide I having the structure as Figure 2.

Compound 2 exhibited benzene proton signals at 6.64 (br,s), 6.72 (d), and 6.60 (d) ppm in its ^1^H NMR spectrum (400 MHz, in CDCl_3_) spectrum. The remaining signals for compound 2 for isopropyl methyl [(CH_3_)_2_CH‐] at δ 0.92 (6H) ppm. At 5.90 (s) ppm, there was only one methylene di‐oxy signal. Signals were detected at 2.42 ppm as a quartet and 2.65 ppm as a triplet by two methylene (‐CH_2_‐) protons of the side chain. The signals at 5.94 (d), 7.19 (d), and 6.14 (m) ppm verified the existence of four methine (–CH‐) protons in the side chain. The last methylene (‐CH_2_‐) next to the nitrogen produced a signal for two protons at 3.57 (t) ppm. The quadrupole broadening at 1.81 ppm indicated the presence of a nitrogen atom.

The presence of an aromatic ring was confirmed by the ^13^C NMR spectrum (100 MHz, in CDCl_3_) of compound 2. Two downfield signals were observed at 147.59, and 145.74 ppm from two oxygenated carbon atoms in the benzene ring, which were found to be different from other carbon atoms in the ring. A signal was detected at 135.17 ppm for the benzene ring's quaternary carbon atom. The remaining three carbons of the benzene ring were methine (‐CH‐) carbons, with signals detected at 108.84, 108.17, and 119.03 ppm.

The signals were detected at 121.19, 141.02, 129.48, and 142.63 ppm for the other four methine (‐CH‐) carbons in the long side chain that are connected to one another by a double bond. The presence of methylene di‐oxy (‐OCH_2_O‐) groups linked to the benzene ring was shown by the signal at 100.8 ppm. At 165.68 ppm, a carbonyl group signal was detected. The methylene (‐CH_2_‐) carbon next to nitrogen was detected at 45.85 ppm for the nitrogen atom. The Lessaigne test revealed that nitrogen was present.

There were two methylene group (‐CH_2_‐) carbons in the side chain, with signals at 35 ppm. The indications of two ‐CH_3_ carbons at 24.65 ppm and one CH carbon at 29.71 ppm indicated the presence of an isopropyl [(CH_3_)_2_CH‐] group in compound 2.

From the physical characteristics and spectral analysis (^1^H‐NMR and ^13^C‐NMR) data of compound 2 and comparing the reported value of ^1^H‐NMR and ^13^C‐NMR spectral data of Chingchengenamide A, the structure of compound 2 was established as Chingchengenamide A having the structure as Figure 2.

### 3.2 Effect of Chabamide I and Chingchengenamide A on Disk Diffusion Assay

**FIGURE 3:**
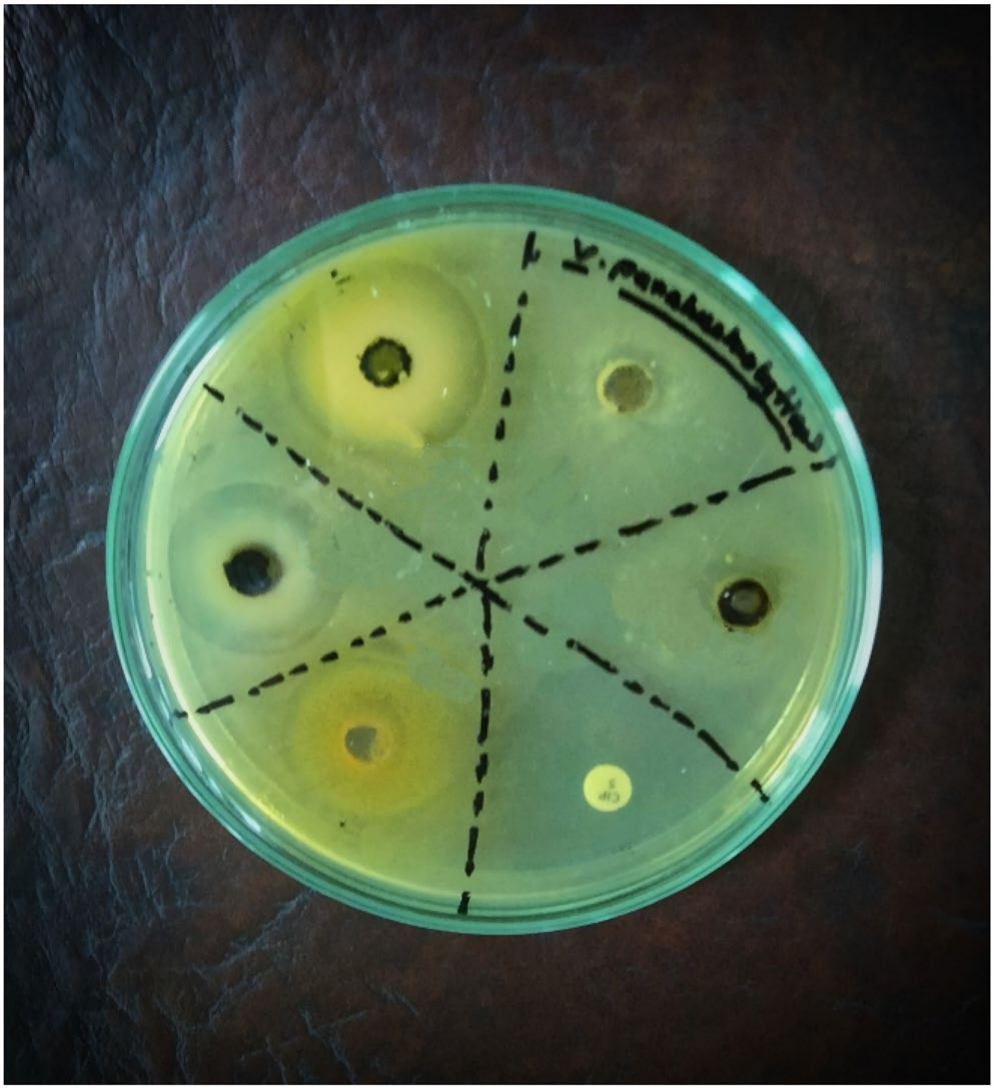
Antimicrobial efficacy evaluation (disk diffusion method) of Chabamide I and Chingchengenamide A along with the standards.

Chabamide I and Chingchengenamide A compounds were assayed for antimicrobial activities against five gram‐positive bacteria and seven gram‐negative bacteria. As a reference standard azithromycin, amoxicillin, and ciprofloxacin were taken for testing the respective antimicrobial activity (Figure 3). The zone of inhibition (ZOI) of the test samples ranged from 11 mm to 21 mm, summarized in Table 2. As per ZOI, the test compound Chabamide exerted notable antimicrobial activity against *Bacillus cereus* , *Pseudomonas aeruginosa* , and *Salmonella typhi* , whereas Chingchengenamide showed considerable activity against *Pseudomonas aeruginosa* with a ZOI of 21 mm.

**TABLE 2:** Antimicrobial activity of Chabamide I and Chingchengenamide A along with standards against gram‐positive and gram‐negative bacterial strains.

Test microorganisms	Zone of Inhibition (mm)				
	Azithromycin (30 μg/disk)	Amoxicillin (30 μg/disk)	Ciprofloxacin (30 μg/disk)	Chabamide I (100 μg/disk)	Chingchengenamide A (100 μg/disk)
Gram‐positive bacteria					
*Bacillus cereus*	35	33	30	20	17
*Bacillus megaterium*	33	29	29	15	14
*Bacillus subtilis*	32	26	33	11	13
*Staphylococcus aureus*	39	36	34	18	15
*Sarcina lutea*	37	32	28	16	16
Gram‐negative bacteria					
*Escherichia coli*	39	37	34	18	17
*Pseudomonas aeruginosa*	42	38	37	21	21
*Salmonella paratyphi*	32	32	26	18	14
*Salmonella typhi*	38	31	35	21	12
*Shigella dysenteriae*	38	30	34	18	15
*Vibrio mimicus*	33	28	27	15	16
*Vibrio parahemolyticus*	40	35	34	16	15

### 3.3 Effect of Chabamide I and Chingchengenamide A on Castor Oil‐Induced Diarrhea

**FIGURE 4:**
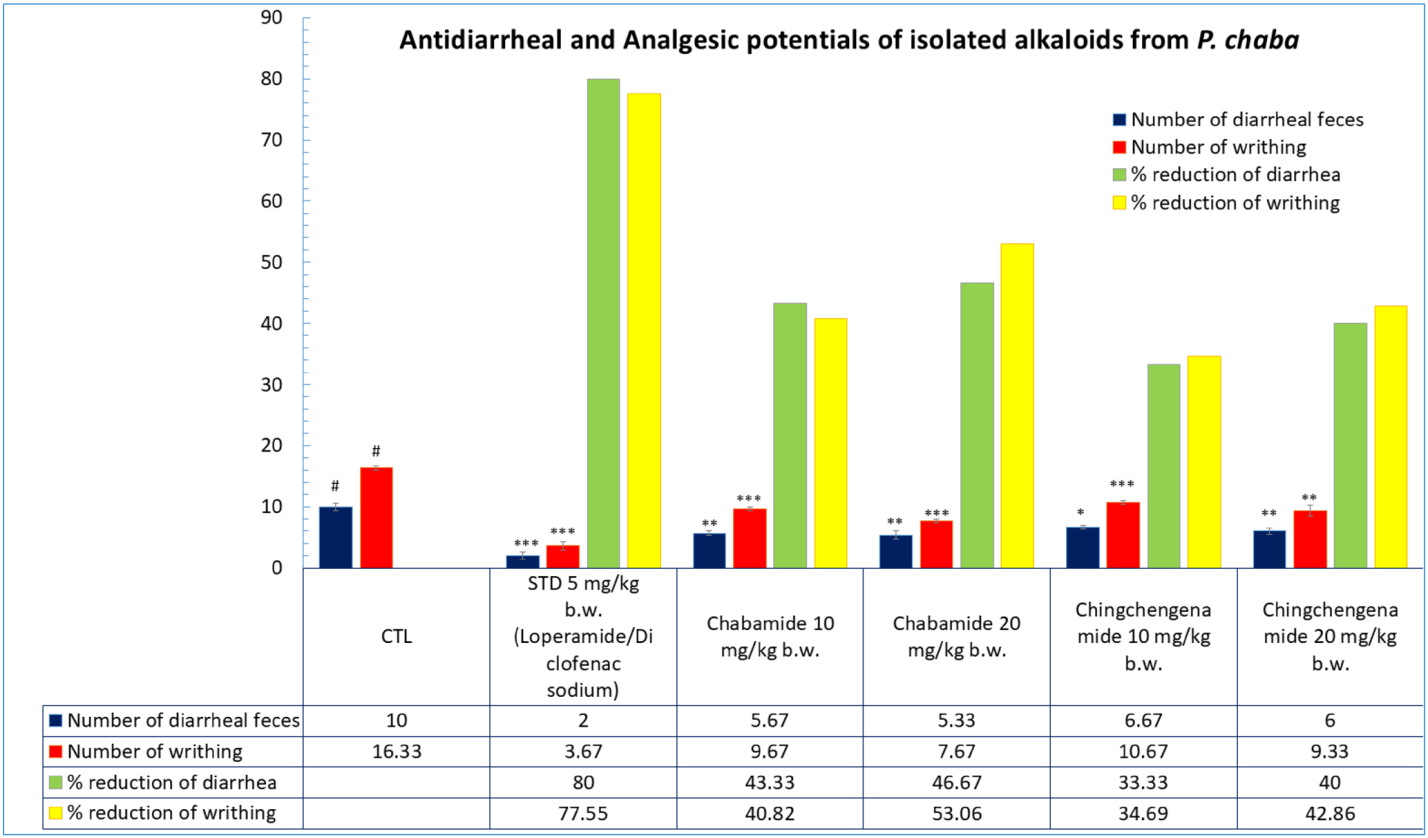
Antidiarrheal and analgesic effect of Chabamide I and Chingchengenamide A along with the standards respectively on castor oil‐induced and acetic acid‐induced tests in mice (values are expressed as Mean ± SEM (*n* = 5); CTL, negative control; STD, positive control; ***p < 0.001, **p < 0.01, *p < 0.05 compared with control and negative control).

Chabamide I and Chingchengenamide A at 10 and 20 mg/kg doses exhibited significant (*p* < 0.05, *p* < 0.01, *p* < 0.001) reduction in the number of feces (Figure 4). In terms of wet feces number, Chabamide I and Chingchengenamide A demonstrated percentages of diarrhea inhibition respectively by 43.33% and 33.33% at 10 mg/kg dose, whereas at 20 mg/kg dose, the values were 46.67% and 40%, respectively, and whereas the value of the standard loperamide was 80%.

### 3.4 Effect of Chabamide I and Chingchengenamide A on Acetic Acid‐Induced Writhing in Mice Model

Chabamide I and Chingchengenamide A at 10 and 20 mg/kg both of the doses exhibited significant (*p* < 0.05, *p* < 0.01, *p* < 0.001) analgesia with a considerable percent reduction of acetic acid‐induced writhing compared with the standard diclofenac sodium (Figure 4). Chabamide exhibited dose‐dependent percent reduction by 40.82% (at 10 mg/kg) and 53.06% (at 20 mg/kg), also together with Chingchengenamide by 34.69% (at 10 mg/kg) and 42.86% (at 20 mg/kg) when compared to the standard with 77.55% reduction.

### 3.5 Molecular Docking Study

#### 3.5.1 MEP Analysis

**FIGURE 5:**
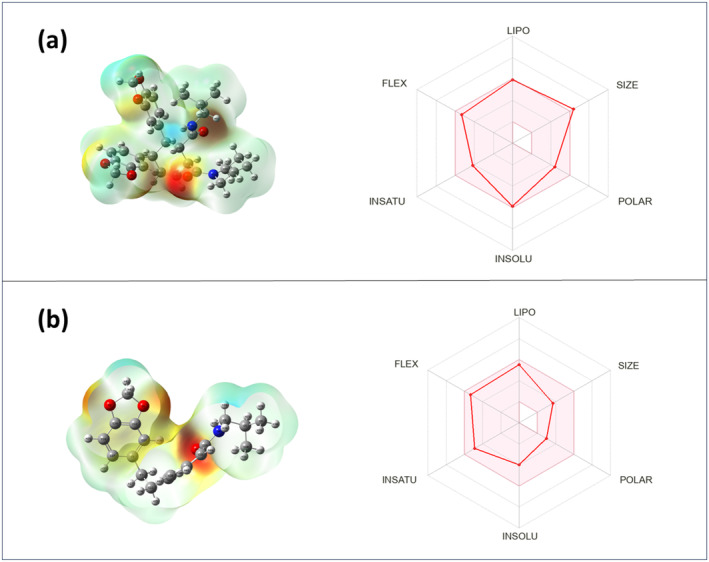
Molecular electrostatic potential map of isolated compounds of *P. chaba*, and boiled egg figures regarding the ADMET analysis of isolated compounds of *P. chaba*, where (a) denotes Chabamide I and (b) denotes Chingchengenamide A.

The MEP analysis (Figure 5) indicates that hydrogen atoms have the highest positive potentiality, and oxygen atoms have the largest negative potentiality. Chabamide I has the highest positive potentiality in our investigation, indicating the largest potential for nucleophilic attack. Furthermore, the highest negative potentiality for Chingchengenamide A indicates the most potential for an electrophile attack.

#### 3.5.2 Molecular Docking and Nonbonded Interactions

Without considering the ligand‐receptor interaction or the chemical or physical properties of receptors and ligands, molecular topology has proven to be a valuable tool for molecular drug design. It was transitioning from an appealing possibility to a cornerstone in the search and development of new drugs. It can be used as a distinct drug design and development paradigm to identify new hits and leads without the need for geometric or physical magnitudes (Zanni et al. 2015). It can be used to construct an effective QSAR method based on topological variables. It helps in the rapid and inexpensive discovery of drugs with superior pharmacodynamic, pharmacokinetic, and toxicological qualities compared with those currently on the market. It can be utilized to determine the mode of action behind a drug's therapeutic effect in conjunction with quantum‐mechanical calculations, docking, and molecular dynamics simulation (Amigó, Gálvez, and Villar 2009). It also enables the discovery of new lead compounds with minimal information acquired from mathematical topological patterns and the interpretation of results in structural and physicochemical terms. As a result, a direct connection between chemical structures and experimental qualities can be made (Diudea, Gutman, and Jantschi 2001).

**FIGURE 6:**
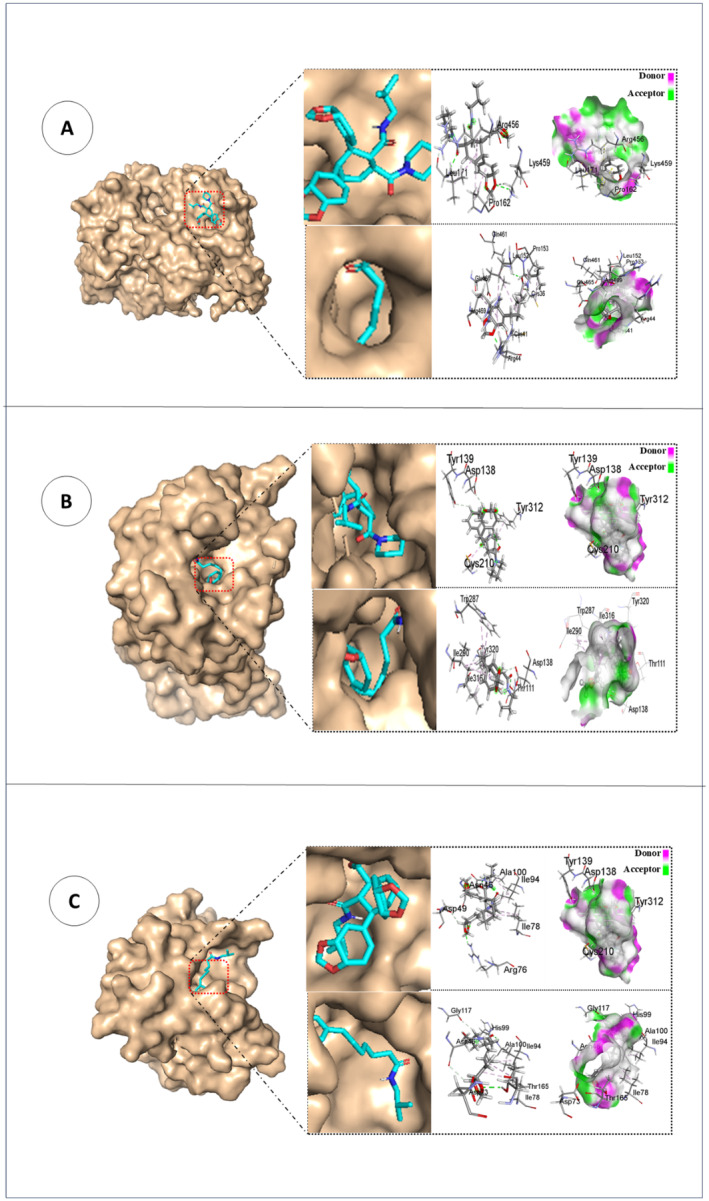
Docked conformation, nonbonding interactions, and hydrogen‐bond surface area of isolated compounds of *P. chaba* at the inhibition binding site of the receptor protein (a) 6COX (analgesic), (b) 6VI4 (antidiarrheal), and (c) 1AJ6 (antimicrobial).

Nonbonding interactions with docking score were utilized as the consideration for the binding affinity and interactions of the isolated compounds from *P. chaba*, along with several standard drugs, including diclofenac, loperamide, ciprofloxacin, amoxicillin, and azithromycin. In the case of the analgesic assay, we investigate the interactions between isolated compounds and prostaglandin‐2 and aspirin‐acetylated human cyclooxygenase‐2 receptor protein. According to our findings, the most significant binding affinities to the prostaglandin‐2 receptor are exhibited by carbamide I (−8.2 kcal/mol), which is greater than the standard drug diclofenac (−7.7 kcal/mol) and the aspirin‐acetylated human cyclooxygenase‐2 receptor by chingchengenamide A (−7.4 kcal/mol), which is closer to diclofenac (−8.4 kcal/mol) represented in Table 3. The carbamide I‐6COX complex is stabilized by four hydrogen bonds as well as four hydrophobic contacts involving LEU171, LYS459, LYS459, LEU171, PRO162, PRO162, LEU171, and ARG456 amino acid residues. In contrast, six hydrogen and alkyl bonds are involved in the interaction between Chingchengenamide A and target cyclooxygenase enzyme through ARG44, GLN461, CYS36, ARG469, CYS41, GLU465, ARG44, LEU152, PRO153, ARG469, LEU152, and CYS36 amino acid residues. In the case of antidiarrheal activity, the isolated compounds from *p. chaba* in this investigation have been docked against the kappa and delta‐opioid receptors. The isolated compounds' docking scores ranged from −6.4 to 8.6 kcal/mol for the delta‐opioid receptor and from −7.6 to −9.7 kcal/mol for the kappa opioid receptor, which is greater than the standard drug loperamide (−8.7 kcal/mol). It is perceived that Chabamide I forms two hydrophobic contacts with CYS210 and TYR312 amino acid residues, as well as two hydrogen bonding interactions with TYR139 and ASP138 amino acid residues. Chingchengenamide A‐kappa opioid receptor complex is stabilized by multiple interactions involving alkyl, pi‐alkyl, and pi‐sulfur interactions as well as hydrogen bonds through THR111, ASP138, ASP138, TYR320, ILE290, ILE290, ILE316, TRP287, and TRP287 amino acid residues. The antibacterial protein and beta‐ketoacyl‐acyl carrier protein synthase III were docked with the isolated compounds of *P. Chaba*. Based on the docking score results, Chabamide I was shown to have the highest binding affinity to the 1AJ6 receptor. Their docking score ranges from −6.2 to −8.5 kcal/mol, which is closer to the standard drug amoxicillin (−7.3 kcal/mol), ciprofloxacin (−7.7 kcal/mol), and azithromycin (−9.1 kcal/mol). The docking score ranking was found to be in the following order: Chabamide I > Chingchengenamide A for the antimicrobial effect of this plant. Four hydrogen‐bonded interactions with amino acid residues ASN46, ARG76, ASP49, and ASN46, and five alkyl interactions with amino acid residues ALA100, ILE94, ILE78, ILE94, and ILE78 are observed in the Chabamide I‐1AJ6 complex. ALA100, THR165, HIS99, ASN46, ASP73, GLY117, ILE78, ILE94, and ILE78 are the involved amino acid residues in stabilizing the chingchengenamide A‐1AJ6 complex through hydrogen bonds as well as alkyl interactions. The nonbonded interactions between amino acid residues of the target receptor for analgesia, diarrhea, microbial infection, and the highest docking scored compounds shown in Table 3 and Figure 6 were analyzed further employing the Biovia Discovery Studio Visualizer 2021 program. As hydrophobic interactions are the major driving forces involved in ligand‐receptor interactions, these amino acid residues with lower interatomic distances (< 5 Å) form strong bonds; hence, they have a greater binding affinity.

**TABLE 3:** Molecular docking and nonbonded interaction of isolated compounds of *P. chaba*.

Receptor	Compounds name	Binding affinity	Bond	Amino acid residues
Cyclooxygenase 2 (6COX)	C1	−8.2	Conventional hydrogen bond	LEU171, LYS459, LYS459
			Alkyl	PRO162, LEU171, ARG456
	C2	−7.5	Conventional hydrogen bond	ARG44, GLN461, CYS36, ARG469, CYS41, GLU465
			Alkyl	ARG44, LEU152, ARG469, CYS36
	Diclofenac Sodium	−7.7	Conventional hydrogen bond	LYS468, ARG469
			Carbon–hydrogen bond	SER471
			Pi‐Alkyl	LYS468
Kappa opioid receptor (6VI4)	C1	−9.7	Conventional hydrogen bond	TYR139, ASP138
			Alkyl	CYS210
			Pi‐Alkyl	TYR312
	C2	−7.6	Conventional hydrogen bond	THR111, ASP138
			Pi‐Sulfur	TYR320
			Alkyl	ILE290, ILE316
			Pi‐Alkyl	TRP287
	Loperamide	−8.4	Conventional hydrogen bond	ARG202
			Carbon–hydrogen bond	CYS210, ASN122
			Alkyl	VAL108, VAL207, VAL118
			Pi‐Anion	ASP138
			Pi‐Alkyl	VAL134
Antibacterial protein (1AJ6)	C1	−8.5	Conventional hydrogen bond	ASN46, ARG76, ASP49
			Alkyl	ILE78, ALA100, ILE94
	C2	−6.7	Conventional hydrogen bond	ALA100, THR165, HIS99, ASN46, ASP73, GLY117
			Alkyl	ILE78, ILE94, ILE78
	Azithromycin	−9.1	Conventional hydrogen bond	GLY77
			Carbon–hydrogen bond	ASP73
			Alkyl	PRO79
	Ciprofloxacin	−7.7	Conventional hydrogen bond	GLY77, THR165
			Carbon–hydrogen bond	ASN46, GLY119
			Alkyl	ILE94, ILE78
			Pi‐Alkyl	ILE78
	Amoxicillin	−7.3	Conventional hydrogen bond	VAL97, ASN46
			Alkyl	ILE78, PRO79, ILE94
			Pi‐Alkyl	ILE94, ALA100

#### 3.5.3 ADMET Prediction

Both *P. chaba* isolated compounds show a good response for human intestinal absorption (HIA) in the current study. Greater HIA values were detected, indicating that they were quickly absorbed from the gastrointestinal tract (GIT) after being taken orally. According to tabulated ADMET data (Table 4) and (Figure 5), Chingchengenamide A permeates all Caco‐2 cells and shows greater C2P values, suggesting that the molecule is entirely absorbed from the GIT with an elevated permeability coefficient. Both substances react favorably to the blood–brain barrier (BBB), indicating increased central nervous system (CNS) permeability. In this case, Chingchengenamide A does not inhibit p‐glycoprotein, suggesting that changing the pharmacokinetic profile of medications is unlikely. Cytochrome P4502C9 (CYP2C9) enzymes metabolize both separated compounds, and this is the leading cause of the significant difference in the pharmacokinetics of many therapeutically essential medicines. Chingchengenamide A was found to have a higher clearance of 0.334, and both compounds showed a modest inhibitory effect on the human ether‐α‐go‐go related gene (hERG). The LD_50_ values of the isolated *P. chaba* compounds in this study range from 2.712 to 3.365 mol/kg, which is a reasonably low‐risk range.

**TABLE 4:** ADMET predictions data of isolated compounds of *P. chaba*.

Name	HIA	C2P	Solubility Log S (ESOL)	Lipophilicity Log Po/w (iLOGP)	BBB	*P*‐GpI	CYP2C9	Clearance	Lipinski Violation	Carcinogens	Acute Oral Toxicity	HERG Inhibition	Rat LD50 (mol/kg)
Chabamide I	1	−0.55	MS (−5.89)	4.18	0.905	I (0.8730)	NI (0.6181)	0.04	1; MW > 500	NC (0.8213)	III (0.7133)	WI (0.9309)	3.365
Chingchengenamide A	1	0.5851	S (−4.00)	3.67	0.9788	NI (0.5372)	I (0.5713)	0.334	0	NC (0.8624)	III (0.6087)	WI (0.9808)	2.712

## 4 Discussion

Bioactive phytochemicals with diverse pharmacological characteristics are abundant in medicinal plants. Determining a medicinal plant's bioactive profile is a well‐established method that involves the extraction and identification of phytochemicals. Various chromatographic techniques, including GC, TLC, HPLC, PTLC, and CC, can be employed in phytochemical analysis to isolate bioactive chemicals from plant extracts. In our investigation, we used ethanol to extract *P. chaba* stems, and then we used the TLC analysis, column chromatographic technique, and PTLC analysis. Two amide alkaloids Chabamide I and Chingchengenamide A were isolated from *P. chaba* stem extracts. Alkaloids are an intriguing class of substances that have a variety of effects on human and animal bodies, both beneficial and harmful. Alkaloids have a wide range of physiological effects, including anticancer, antibacterial, antimitotic, hypnotic, anti‐inflammatory, antitumor activity, analgesic, psychotropic, local anesthetic properties, and many others (Adamski et al. 2020). Chingchengenamide A was a derivative of piperine. Structure–activity relationship showed that this compound has greater biological activity than piperine (Shinta, Choodej, and Pudhom 2021). In the case of amide alkaloids, double bonds in the side chain will not be responsible for biological activity. Structure–activity relationship study recommended that both alkaloids Chabamide I and Chingchengenamide A demonstrated inhibitory effectiveness against at least one of eight tested human tumor cell lines. The 3,4,5‐trimethoxy substitution in the phenyl ring of amide alkaloids improved selectivity against MDR subline KBvin. However, the 4‐methoxy group in this pattern is necessary for cytotoxic action (Wang et al. 2014).

The current study revealed the isolation of two alkaloids from *P. chaba* including Chabamide I and Chingchengenamide A. According to the best of our knowledge, Chingchengenamide A is isolated from *P. chaba* stems for the first time. This alkaloid was isolated for the first time from the China piper species *Piper otonoids* (Gómez‐Calvario et al. 2019). On the contrary, Chabamide I was isolated earlier from *P. chaba* (Rukachaisirikul et al. 2002).

Instances of inflammation, such as microbial intrusion or tissue trauma, elicit the recruitment of defensive leukocytes to the affected site, a meticulously coordinated response mediated by receptors like toll‐like receptors (TLRs) and NOD‐like receptors (NLRs) (Abdel Motaal and Abdel Maguid 2005). Concurrently, an extensive array of inflammatory mediators, including eicosanoids, cytokines, chemokines, and vasoactive amines, are unleashed, contributing to the intricate orchestration of inflammatory cascades (Sun, Sit, and Feinberg 2014). Previous research indicates that alkaloids possess the capability to inhibit phospholipase A2, consequently reducing the accessibility of arachidonic acid, a precursor essential for prostaglandin synthesis (Hayfaa, Sahar, and Awatif 2013). Plant extracts rich in alkaloids have been noted to modulate the activity of cyclooxygenase 1 and 2 (COX‐1 and COX‐2), which are used to produce inflammatory mediators. (Mishra, Seth, and Maurya 2016).

Additionally, some researchers have theorized that the analgesic effects of plant extracts may stem from their ability to suppress the release of interleukin‐1β and interleukin‐8 by resident peritoneal cells or to hinder the production of prostaglandins and bradykinin (Hayfaa, Sahar, and Awatif 2013). Both Chabamide I and Chingchengenamide A showed prospective analgesic activity in the mice model though chabamide showed relatively better outcomes, which ascertains a better prospect of dimeric alkaloid as an analgesic drug candidate.

Castor oil, esteemed for its potent laxative attributes, functions as a proficient inducer of diarrhea owing to its hydrolyzation in the upper small intestine, resulting in the generation of ricinoleic acid. This compound orchestrates fluid secretion while concurrently impeding water and electrolyte absorption, alongside diminishing active Na^+^ and K^+^ absorption and Na^+^, K^+^, ^−^ATPase activity in both the small intestine and colon (Ammon, Thomas, and Phillips 1974). These effects are ascribed to the irritant properties of ricinoleic acid, released upon exposure to pancreatic acid (Mbagwu and Adeyemi 2008). Additionally, ricinoleic acid elicits the release of prostaglandins, pivotal regulators of gastrointestinal function, thereby instigating motility and secretion, culminating in diarrhea (Hu et al. 2009; Agunu et al. 2005). Recent elucidations concerning molecular mechanisms propose the activation of the EP3 prostanoid receptor by ricinoleic acid, thereby mediating the pharmacological effects of castor oil. This receptor activation in intestinal and uterine muscle cells elucidates the intricate cellular and molecular pathways underlying castor oil's laxative action. Furthermore, the antidiarrheal efficacy of two isolated alkaloids may operate through diverse mechanisms, including the modulation of prostaglandin secretion (Tunaru et al. 2012). Chabamide I displayed a more potent action compared with Chingchengenamide A. From this investigation, it can be postulated that dimeric alkaloid is a better candidate than monomeric leads. At 20 mg/kg b.w. dose, both alkaloids showed better results than 10 mg/kg b.w. dose despite the absence of a dose‐dependent pattern.

Antibacterial agents possess the capacity to disrupt the structural integrity of bacterial cell walls, resulting in the leakage of cytoplasmic contents and subsequent coagulation, particularly notable in gram‐positive bacterial strains. Analogously, phytochemical constituents identified within the methanol extract of *C. gigantea* leaves are speculated to intercede in various bacterial biosynthetic pathways, potentially functioning as inhibitors of cell wall, DNA, lipid, and/or protein synthesis (Frassinetti et al. 2020). The principal antibacterial mechanisms attributed to alkaloids encompass the inhibition of bacterial cell wall synthesis, alteration of cell membrane permeability, suppression of bacterial metabolism, and interference with nucleic acid and protein synthesis (Yan et al. 2021). Unlike the antidiarrheal effect, both Chabamide I and Chingchengenamide A demonstrated noteworthy antibacterial efficacy against gram‐positive and negative bacteria compared with standards.

Molecular docking can be employed to screen great libraries of bioactive chemicals involved in modifying the activity and function of a biological protein. It is now a valuable tool for predicting the shape of the ligand‐target complex, identifying new ligands and chemical probes, and improving hit rates (Agu et al. 2023). To determine the category of nonbonded interactions underlying the stability of compound‐target complexes, all isolated compounds were docked with the active site of diverse receptors, such as prostaglandin‐2, aspirin‐acetylated human cyclooxygenase‐2, delta‐opioid receptor, kappa opioid receptor, beta‐ketoacyl‐acyl carrier protein synthase III, and antibacterial protein. The active compounds (C1‐C2) of *P. Chaba* had docking scores of −7.3, −7.4, −7.5, −8.2, −8.5, and − 9.7 kcal/mol to the chosen target, in that order. Developing nonbonding interactions, such as hydrogen bonds, hydrophobic interactions, and van der Waals interactions with major amino acids, is a crucial phase in ligand docking in favorable conformations, as indicated by the docking score. When the docking poses are identified using the Pyrx Virtual Screening tool, the results show that conventional hydrogen bonds, carbon–hydrogen bonds, pi‐alkyl, alkyl, and pi‐sulfur primarily involve interactions between isolated compounds and selected targets. The molecular ESP depicts the molecules' electrical distribution and nuclear charge in their immediate surroundings. Additionally, it deliberates electronegativity, dipole moment, molecular characteristics, chemical reactivity, and intermolecular interaction (Murray and Politzer 2011). Hydrogen bonding and biological recognition are both explained by MEP. It can be helpful when examining the reaction locations for nucleophilic and electrophilic attacks on the chosen molecules. The most negatively charged areas are indicated by a deep red color, which suggests an electron‐rich area that would be perfect for an electrophilic attack. On the contrary, dark blue represents the most advantageous feature, which is suited for nucleophilic assault as it is electron‐free (Weiner et al. 1982). In the early stages of drug design and development, ADMET analysis is a key focus to exclude subpar substances based on their pharmacokinetic profile, which includes absorption, distribution, metabolism, excretion, and toxicity (Davis and Riley, 2004). AdmetSAR uses a 0–1 scale to quantify the Admet scores of 18 different qualities; a 1 indicates the best, and a 0 indicates poisonous or detrimental. The human intestinal epithelial Caco‐2 cell has developed into a gold standard model for predicting HIA of drugs, apparent permeability coefficients, the role of intestinal CYP isozymes in metabolism, and intestinal phase II drug‐metabolizing enzymes. This model makes early drug development more efficient and repeatable (Van Breemen and Li 2005). The CNS is not protected from the negative effects of ligands because they can all easily pass across the BBB (Nisha et al. 2016). Depending on its roles, the p‐glycoprotein substrate can act as an inducer or an inhibitor. Inducing p‐glycoprotein would decrease the drug's bioavailability. However, p‐glycoprotein inhibition may affect a drug's pharmacokinetic characteristics (Ahmed Juvale et al. 2022). Chingchengenamide A metabolic CYP2C9 enzyme inhibitory function increases the likelihood of drug–drug interactions, which may significantly alter the pharmacokinetic profile of pharmaceuticals. As chabamide I is not engaged in CYP2C9 inhibition, there is little chance that the pharmacokinetic profile of medications or the interactions that accompany them would change (Shirasaka et al. 2013). In postmarketing surveillance, the main cause of cardiotoxicity such as long QT syndrome (LQTS), ventricular fibrillation, ventricular arrhythmia, and sudden death is inhibition of the human ether‐α‐go‐go‐related gene (hERG), which encodes the voltage‐gated potassium channel (Kv11.1) (Creanza et al. 2021). One compound's computed molecular weight of more than 500 g/mol indicates that it breaches the RO5 criterion, according to the SwissADME computed data. This RO5 rule mostly applies to medications passively absorbed across cell membranes. Adverse consequences arising from acute oral toxicity suggest that chemicals affect living things, including people, through various biochemical processes. Acute cutaneous, inhalation, and oral rodent toxicity are crucial factors to consider when assessing substances' toxicological effects (Lagunin et al. 2011). Moreover, both substances exhibit noncarcinogenicity and acute oral toxicity in category III, indicating their comparatively reduced toxicity. For rodent acute toxicity, the median lethal dose (LD_50_) is a commonly used method for categorizing substances according to their likely toxicity to human health after direct exposure. Compared with other conventionally available medications for the treatment of pain, diarrhea, and microbial infection, Chingchengenamide A may be a better potential candidate for the development of novel analgesic, antidiarrheal, and antimicrobial agents due to its excellent docking score, nonbonding interactions with cyclooxygenase‐II enzyme, kappa opioid receptor, and antibacterial protein, as well as its excellent pharmacokinetic characteristics and drug‐likeness screening test.

## 5 Conclusion

From this research work, two compounds were isolated through the phytochemical study of the *P. chaba* stems. These compounds were identified by spectroscopic techniques (^1^H and ^13^C NMR) as Chabamide I and Chingchengenamide A. The separated plant metabolites may have therapeutic value. The analgesic, antibacterial, and antidiarrheal qualities of isolated phytochemicals were significant. Computer‐simulated molecular docking and ADME/T analysis also confirmed that isolated compounds of *P. chaba* have high affinity with selected targets as well as good efficacy. The results of this pioneering study provide a scientific basis for the traditional uses of *P. chaba* as a remedy for various complications and potential leads for novel drug discovery and therapeutic advancement. However, more investigation is required to ascertain more secondary metabolites from *P. chaba* that may be in charge of more extensive bioactivities. In addition, clinical research is still recommended in light of the available data to confirm the plant's purported pharmacological properties along with their safety, efficacy, and toxicological parameters.

## Author Contributions

**Shabiba Parvin Shandhi:** conceptualization (lead), formal analysis (equal), investigation (lead), software (equal), supervision (equal), validation (equal), visualization (equal), writing – original draft (equal). **Fahmida Tasnim Richi:** conceptualization (equal), data curation (equal), formal analysis (lead), investigation (equal), methodology (lead), resources (equal), software (equal), validation (equal). **Safaet Alam:** conceptualization (lead), data curation (lead), investigation (equal), methodology (equal), software (equal), supervision (equal), visualization (equal). **Kutub Uddin Ahamed:** conceptualization (equal), data curation (equal), formal analysis (equal), methodology (equal), software (lead), writing – review and editing (equal). **Nazim Uddin Emon:** conceptualization (equal), data curation (equal), formal analysis (equal), resources (equal), software (equal). **Najneen Ahmed:** conceptualization (equal), data curation (equal), investigation (equal), methodology (equal), resources (equal), software (equal), validation (equal). **Chuxiao Shao:** data curation (equal), formal analysis (equal), investigation (equal), software (equal), supervision (equal), visualization (equal), writing – original draft (equal), writing – review and editing (equal). **Shuanghu Wang:** data curation (equal), formal analysis (equal), methodology (equal), resources (equal), software (equal), validation (equal), visualization (equal), writing – original draft (equal), writing – review and editing (equal). **Peiwu Geng:** formal analysis (equal), methodology (equal), software (equal), validation (equal), visualization (equal), writing – original draft (equal), writing – review and editing (equal). **Abdullah Al Mamun:** data curation (equal), formal analysis (equal), funding acquisition (lead), investigation (equal), resources (equal), software (equal), supervision (lead), validation (lead), visualization (lead), writing – review and editing (lead).

## Conflicts of Interest

The authors declare no conflicts of interest.

## Supporting information


**Figure S1.** The ^1^H‐NMR spectrum (400 MHz, CDCl_3_) of compound 1 (Chabamide I).
**Figure S2**. The ^13^C NMR spectrum (100 MHz, in CDCl_3_) of compound 1 (Chabamide I).
**Figure S3**. The ^1^H‐NMR spectrum (400 MHz, CDCl_3_) of compound 2 (Chingchengenamide A).
**Figure S4**. The ^13^C NMR spectrum (100 MHz, in CDCl_3_) of compound 2 (Chingchengenamide A).

## Data Availability

The data that support the findings of this study are available from the corresponding author upon reasonable request.
