# Supplementary material for: Isolation, Structure Elucidation, and Bioactivity Evaluation of Two Alkaloids From Piper chaba H. Stem: A Traditional Medicinal Spice and Its Chemico‐Pharmacological Aspects
Source: Food Sci Nutr. 2024 Nov 22;12(12):10680–98. doi: 10.1002/fsn3.4585 (PMC11666819; doi:10.1002/fsn3.4585)
Supplement: Supplementary file 1 — Figure S1. The 1H‐NMR spectrum (400 MHz, CDCl3) of compound 1 (Chabamide I). Figure S2. The 13C NMR spectrum (100 MHz, in CDCl3) of compound 1 (Chabamide I). Figure S3. The 1H‐NMR spectrum (400 MHz, CDCl3) of compound 2 (Chingchengenamide A). Figure S4. The 13C NMR spectrum (100 MHz, in CDCl3) of compound 2 (Chingchengenamide A). [file FSN3-12-10680-s001.docx]

**Supplementary Figures**


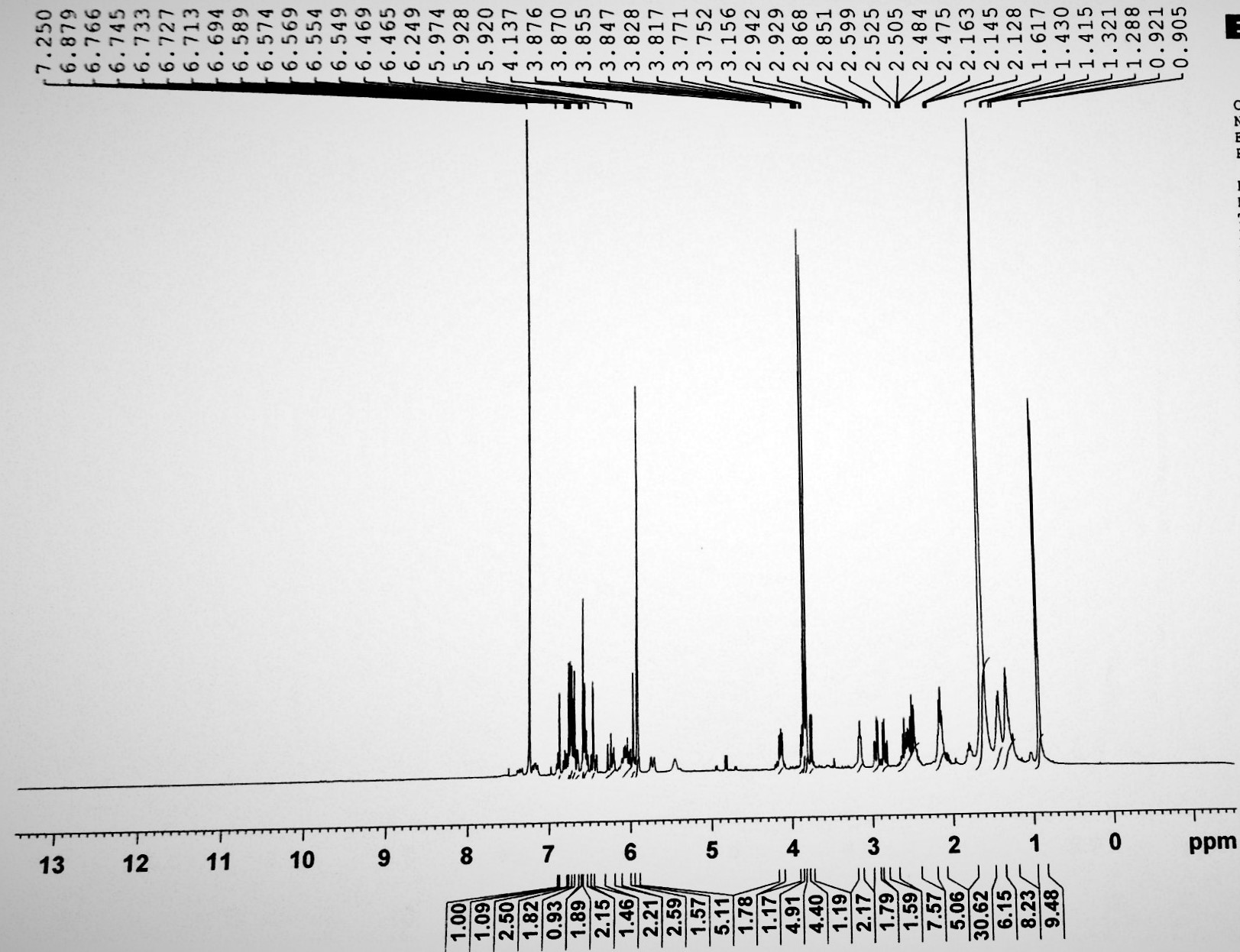


**Supplementary Figure 1:** The ^1^H-NMR spectrum (400 MHz, CDCl_3_) of compound 1 (Chabamide I)


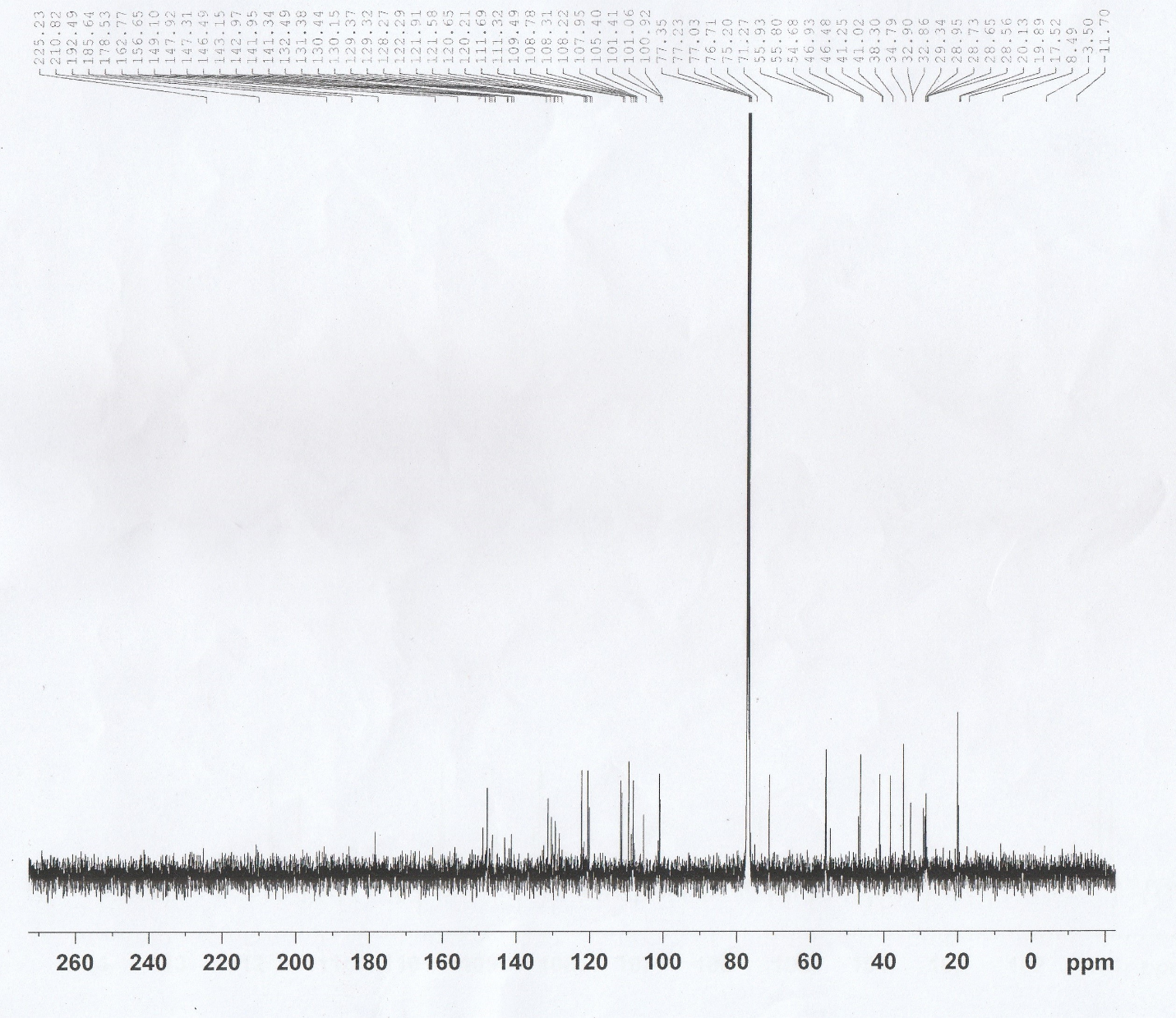


**Supplementary Figure 2:** The ^13^C NMR (100 MHz, in CDCl_3_) spectrum of compound 1 (Chabamide I)


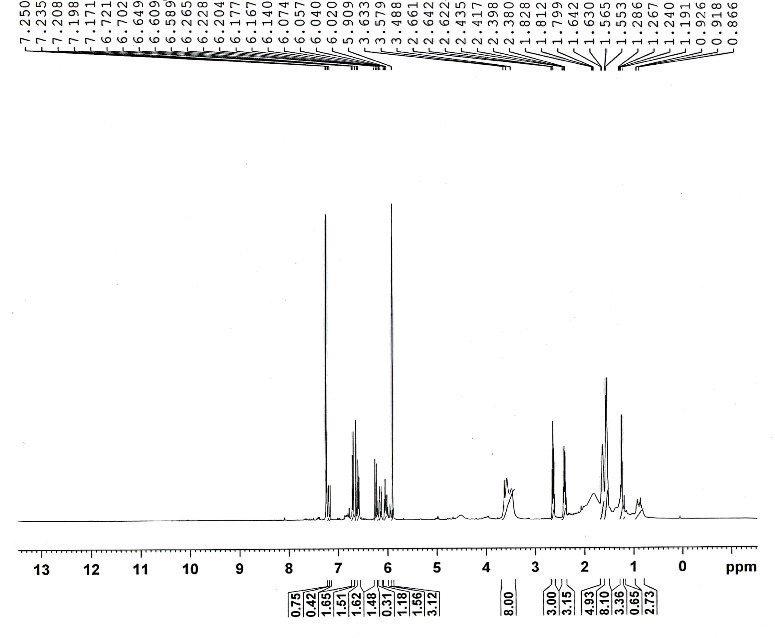


**Supplementary Figure 3:** The ^1^H-NMR spectrum (400 MHz, CDCl_3_) of compound 2 (Chingchengenamide A)


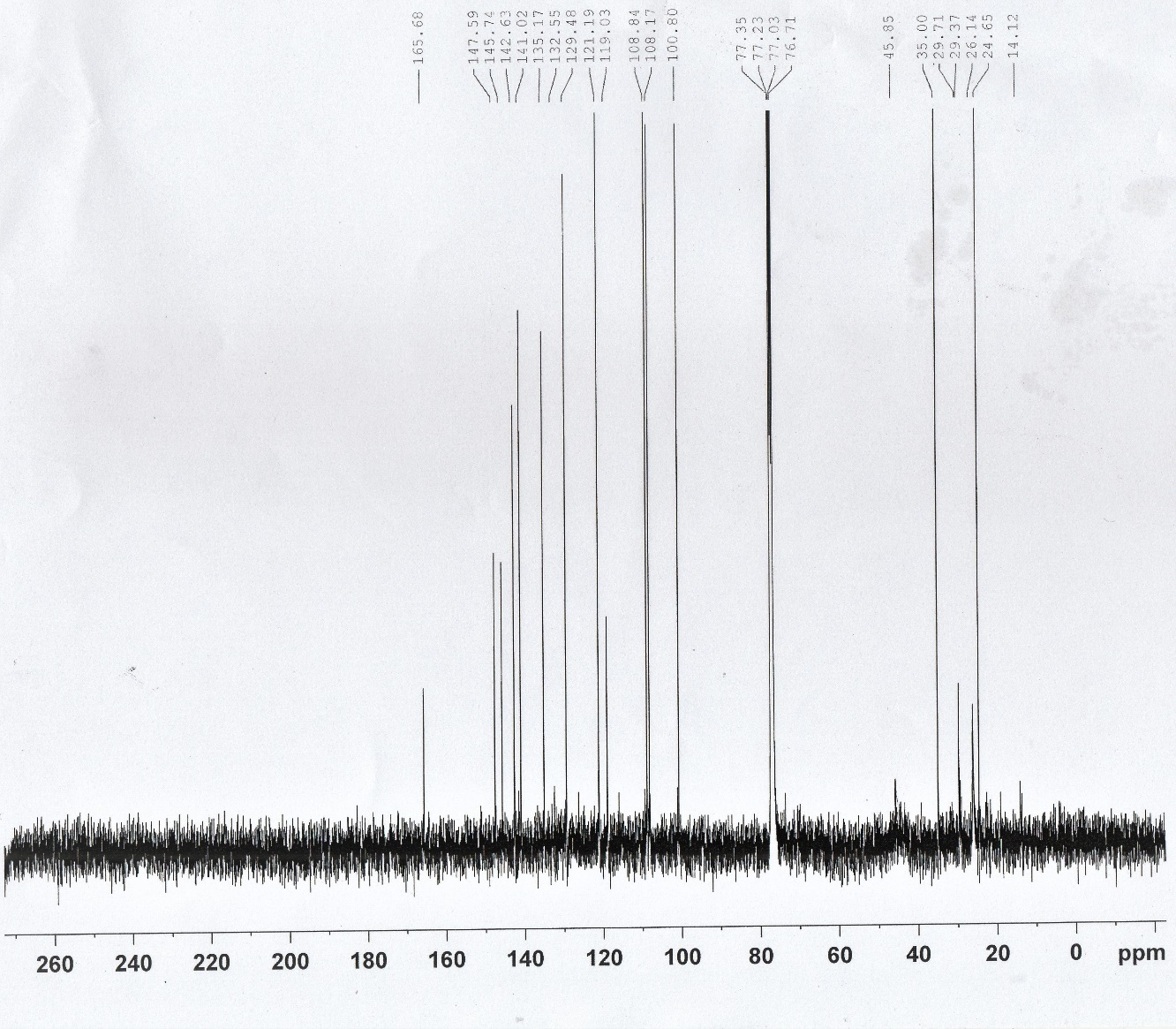


**Supplementary Figure 4:** The ^13^C NMR (100 MHz, in CDCl_3_) spectrum of compound 2 (Chingchengenamide A)
